# Immune Checkpoint Inhibitor-Induced Sarcoidosis-Like Reaction Mimicking Pulmonary Metastasis: A Case Report

**DOI:** 10.7759/cureus.107995

**Published:** 2026-04-29

**Authors:** Mari Sawada, Yoshiko Kurose, Yoshifumi Ochi, Kiyoshi Kanno, Masaaki Andou

**Affiliations:** 1 Department of Gynecology, Kurashiki Medical Center, Kurashiki, JPN

**Keywords:** immunotherapy-related adverse events, pembrolizumab, pulmonary nodules, sarcoidosis-like reaction, uterine carcinosarcoma

## Abstract

Immune checkpoint inhibitors can induce sarcoidosis-like reactions that mimic disease progression and pose diagnostic challenges. We report a case of a 77-year-old woman who developed pulmonary nodules during pembrolizumab therapy for recurrent uterine carcinosarcoma. Despite radiologic suspicion of metastatic disease, systemic tumor control was maintained. Thoracoscopic wedge resection revealed non-caseating granulomas without evidence of malignancy or infection, confirming a sarcoidosis-like reaction. Pembrolizumab therapy was continued without corticosteroid treatment, and the patient remained disease-free three years after treatment completion. This case highlights an important diagnostic pitfall during immune checkpoint inhibitor therapy. Sarcoidosis-like reaction should be considered in the differential diagnosis of newly developed pulmonary lesions, and histologic confirmation is essential to avoid misdiagnosis and inappropriate discontinuation of effective immunotherapy.

## Introduction

Immune checkpoint inhibitors have significantly improved outcomes in a variety of malignancies by enhancing antitumor immune responses. These agents block inhibitory immune checkpoints such as programmed death-1 and programmed death ligand-1, thereby restoring T-cell-mediated antitumor immunity [1]. Immune checkpoint inhibitors have demonstrated significant clinical activity in tumors characterized by microsatellite instability-high or mismatch repair deficiency, including gynecologic malignancies such as endometrial cancer [2]. Clinical trials such as the KEYNOTE-158 study have demonstrated durable antitumor activity of pembrolizumab in patients with microsatellite instability-high advanced endometrial cancer [3]. However, immune checkpoint inhibitors are also associated with various immune-related adverse events caused by immune activation, which may involve multiple organ systems.

Sarcoidosis-like reaction is a rare immune-related adverse event characterized by the formation of non-caseating granulomas in affected tissues [4]. It may present with pulmonary nodules or lymphadenopathy that resembles metastatic disease, and differentiation from tumor progression can be challenging during immune checkpoint inhibitor therapy. Here, we report a case of pulmonary drug-induced sarcoidosis-like reaction occurring during pembrolizumab treatment for recurrent uterine carcinosarcoma. This case highlights a key diagnostic pitfall during immune checkpoint inhibitor therapy.

## Case presentation

**Figure 1:**
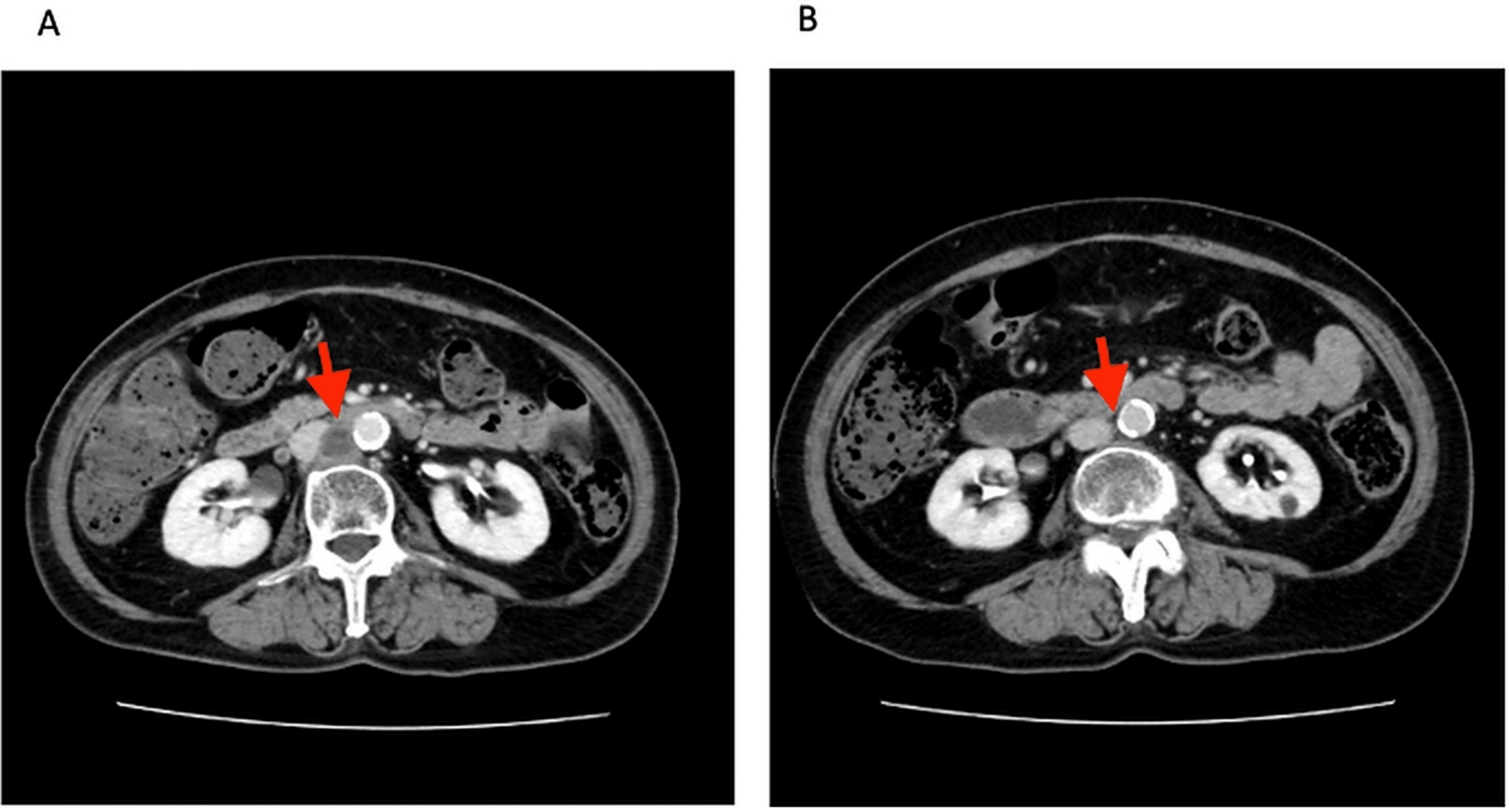
Computed tomography (CT) findings for the response to pembrolizumab treatment. (A) CT demonstrating multiple enlarged lymph nodes (red arrow) in the para-aortic regions after adjuvant chemotherapy. (B) Follow-up CT showing complete radiologic response after pembrolizumab therapy (red arrow).

A 77-year-old Asian woman (gravida three, para two) presented with abnormal uterine bleeding. Her surgical history included cesarean delivery, left salpingo-oophorectomy, and left hemicolectomy for colon cancer. Pelvic imaging revealed a 6 cm uterine mass, and she underwent total hysterectomy with right salpingo-oophorectomy and pelvic lymphadenectomy. Histopathology demonstrated uterine carcinosarcoma, International Federation of Gynecology and Obstetrics (FIGO) stage IB (pT1bN0M0). Adjuvant chemotherapy with paclitaxel and carboplatin was administered. After four cycles, chest and abdominal computed tomography revealed multiple lymph node metastases in the common iliac, para-aortic, and mediastinal regions (Figure 1A). Microsatellite instability testing demonstrated microsatellite instability-high status, and pembrolizumab monotherapy was initiated.

**Figure 2:**
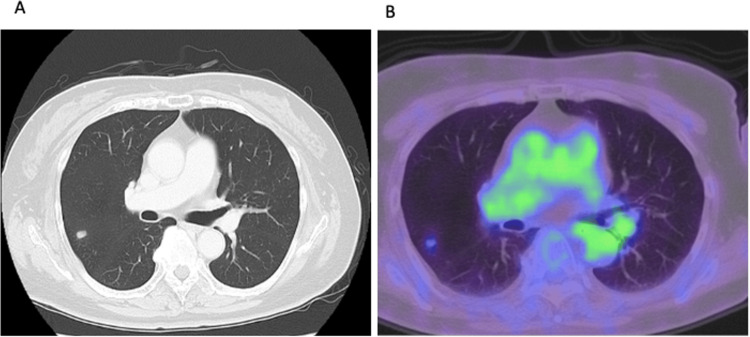
Pulmonary nodule detected during pembrolizumab therapy. (A) Computed tomography (CT) demonstrating a newly developed pulmonary nodule in the right lung. (B) Positron emission tomography (PET) showing mild fluorodeoxyglucose uptake in the right upper lobe lesion without uptake in previously involved lymph nodes.

During pembrolizumab therapy, the patient developed immune-related adverse events including uveitis, hypopituitarism with secondary adrenal insufficiency, and hypothyroidism, all of which were managed appropriately. A complete radiologic response was observed in all previously involved lymph node lesions (Figure 1B). Nevertheless, after 21 cycles of pembrolizumab, newly developed pulmonary nodules were identified on computed tomography. Positron emission tomography demonstrated mild fluorodeoxyglucose uptake in these lesions, raising suspicion of metastatic disease (Figures 2A, 2B).

**Figure 3:**
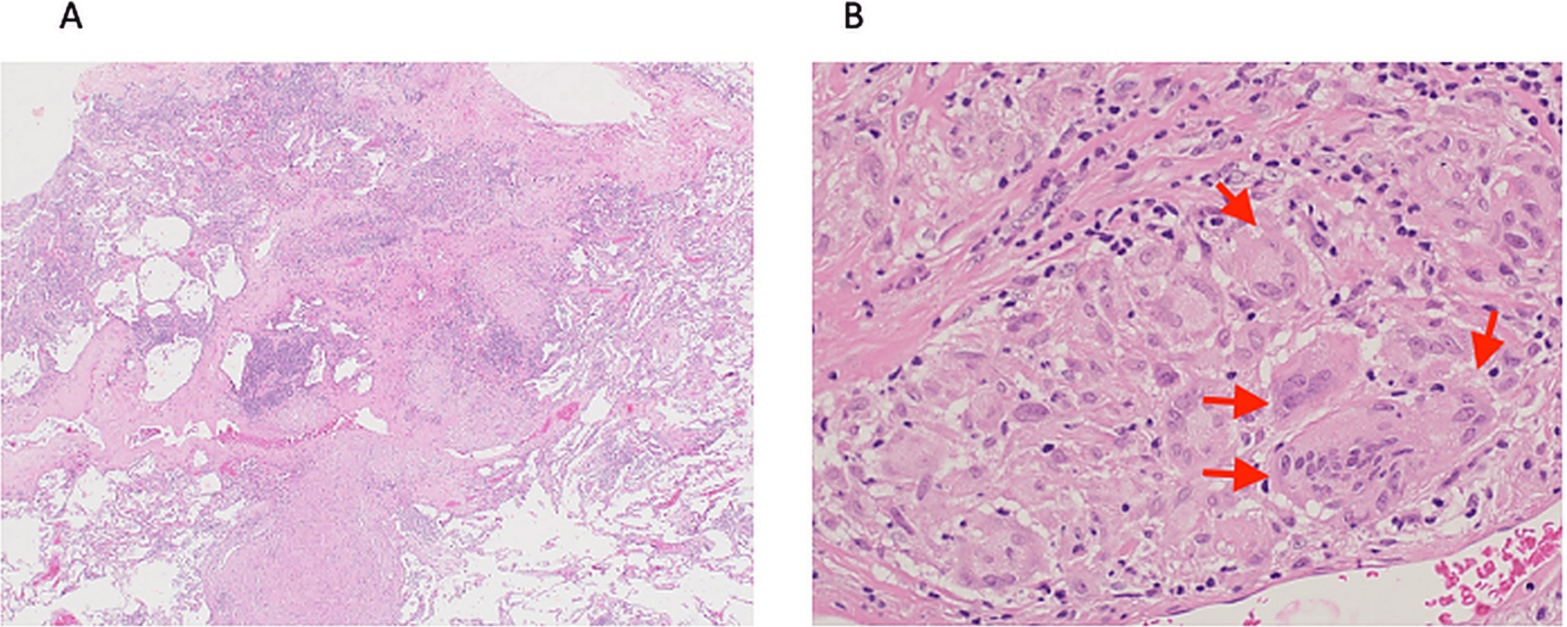
Histopathological characteristics of sarcoidosis-like reaction (hematoxylin-eosin staining). (A) Low-magnification view showing non-caseating epithelioid granulomas, 100x. (B) High-magnification view demonstrating epithelioid histiocytes and multinucleated giant cells (red arrows) without evidence of malignancy, 400x.

As the disease appeared confined to the lung, thoracoscopic wedge resection of the right upper and middle lobes was performed. The operative time was 62 minutes, with minimal blood loss and no perioperative complications. Histopathological examination demonstrated non-caseating epithelioid granulomas without evidence of malignancy, and special stains including acid-fast bacilli and Periodic acid-Schiff stain were negative for infectious organisms, supporting the diagnosis of drug-induced sarcoidosis-like reaction, a rare immune-related adverse event (Figures 3A, 3B). The patient was informed of the diagnosis and consented to the continuation of pembrolizumab under close monitoring. Pembrolizumab therapy was continued and completed for a total of 40 cycles. Three years after treatment completion, the patient remains disease-free, with no recurrence of carcinosarcoma, relapse of pulmonary granulomas, or new immune-related adverse events.

## Discussion

Sarcoidosis is a multisystem granulomatous disease of unknown etiology characterized by the presence of non-caseating granulomas in affected tissues [5]. The disease most commonly affects the lungs but can involve virtually any organ, including the eyes, skin, heart, and lymphatic system. The clinical course is highly variable, ranging from spontaneous remission to chronic progression with significant organ dysfunction [6]. In contrast, drug-induced sarcoidosis-like reaction is defined as a granulomatous reaction temporally associated with exposure to a specific drug [7]. Histopathologic findings are indistinguishable from those of idiopathic sarcoidosis; therefore, diagnosis is primarily based on the temporal relationship between drug exposure and the development of granulomatous lesions. Chopra et al. proposed a classification system categorizing drug-induced sarcoidosis-like reaction as highly likely, probable, or possible based on clinical response following drug withdrawal [7]. In the present case, the response to drug withdrawal could not be evaluated because complete surgical resection of the lesions was performed, and the diagnosis was classified as probable drug-induced sarcoidosis-like reaction.

Immune checkpoint inhibitors have emerged as one of the drug classes associated with drug-induced sarcoidosis-like reaction [7]. Sarcoidosis-like reaction is considered a rare immune-related adverse event and has been reported to occur in approximately one to two percent of patients receiving immune checkpoint inhibitor therapy [4,7]. The underlying mechanism is thought to involve dysregulated immune activation induced by checkpoint blockade. Inhibition of CTLA-4 or PD-1 signaling enhances T-cell activation and promotes a T helper 1-dominant immune response with increased production of interferon-γ and tumor necrosis factor-α, cytokines that play central roles in granuloma formation [4,8].

Initial reports of immune checkpoint inhibitor-associated sarcoidosis-like reactions were primarily described in melanoma patients treated with anti-CTLA-4 antibodies [9,10]. Subsequently, similar reactions have been reported in patients treated with anti-PD-1 agents, including nivolumab and pembrolizumab [11,12]. Sarcoidosis-like reactions have been reported across multiple malignancies treated with immune checkpoint inhibitors, most commonly melanoma and lung cancer [4,13,14]. To our knowledge, reports on gynecologic malignancies remain extremely limited. Nykaza et al. reported two cases of immune checkpoint inhibitor-associated sarcoidosis-like reactions in gynecologic malignancies. One patient with high-grade serous ovarian cancer developed symptomatic mediastinal lymphadenopathy approximately three months after initiation of anti-PD-1 therapy, was diagnosed by biopsy, and continued treatment but ultimately died of disease [14]. The second case involved a patient with peritoneal carcinosarcoma and pre-existing asymptomatic sarcoidosis, who developed mediastinal and hilar lymphadenopathy with pulmonary ground-glass opacities; despite continuation of therapy, the patient also died of disease [14]. In contrast, our patient remained asymptomatic and achieved durable disease control with continued pembrolizumab therapy following histologic confirmation.

Sarcoidosis-like reactions frequently involve mediastinal lymph nodes and pulmonary parenchyma and may mimic metastatic disease. Newly developed pulmonary nodules during immune checkpoint inhibitor therapy do not necessarily indicate tumor progression [4,8]. Misinterpretation may lead to premature discontinuation of effective immunotherapy. Furthermore, the highly variable time to onset further complicates differentiation from disease progression. In a comprehensive review, Gkiozos et al. reported that the median time to onset of immune checkpoint inhibitor-associated sarcoidosis-like reactions was 14 weeks (range, 3-92 weeks), with mediastinal lymph nodes, lungs, and skin representing the most frequently involved sites [4].

The principal differential diagnoses include granulomatous infection and tumor-associated sarcoid reactions. Granulomatous infections typically demonstrate caseating necrosis and identifiable organisms on special stains [6]. Tumor-associated sarcoid reactions have long been recognized in patients with malignancies. They likely reflect immune responses to tumor-associated antigens and are typically confined to tumor-draining lymph nodes or the tumor microenvironment, rather than manifesting as systemic granulomatous disease. Additional reports of sarcoid-like reactions in gynecologic malignancies remain limited. Published cases include ovarian clear cell carcinoma with sarcoid reaction in the spleen and regional lymph nodes, vaginal cancer with mediastinal and hilar lymphadenopathy mimicking recurrence, and uterine cancer with splenic sarcoid reaction after surgery, in which such reactions may mimic recurrence or metastatic disease [15-17]. In the present case, the absence of necrosis, negative microbiologic findings, sustained systemic tumor control, and coexistence of endocrine immune-related adverse events were consistent with a drug-induced immune mechanism, although a definitive causal relationship could not be established.

Histologic evaluation should therefore be considered when imaging findings are inconclusive, particularly in patients who otherwise demonstrate sustained tumor control. Importantly, sarcoidosis-like reactions often exhibit increased fluorodeoxyglucose uptake on positron emission tomography, which may result in false-positive findings, potentially leading to misinterpretation as metastatic disease or tumor progression [18,19]. In the present case, thoracoscopic wedge resection was performed to establish a definitive diagnosis because the pulmonary nodules were peripherally located and considered suboptimal for bronchoscopic biopsy. Histologic confirmation allowed exclusion of metastatic disease and continuation of effective immunotherapy.

Management of drug-induced sarcoidosis-like reaction should be individualized. While drug discontinuation is often recommended [7], several reports have demonstrated that immune checkpoint inhibitor therapy can be continued in selected patients with mild or asymptomatic sarcoidosis-like reactions [4]. Although corticosteroids were administered in approximately half of the reported cases, clinical outcomes were generally favorable, and continuation of immune checkpoint inhibitor therapy was feasible in selected patients without disease progression [4]. Recent data also support this clinical heterogeneity. Smith et al. reported that multisystem sarcoidosis-like reactions were more frequently symptomatic and required corticosteroid therapy and discontinuation of immune checkpoint inhibitors, whereas single-organ involvement was often asymptomatic and could be managed conservatively, with continuation of immunotherapy in selected cases [20]. In our patient, the absence of symptoms and histologic exclusion of metastasis supported continuation of pembrolizumab under careful surveillance. Durable remission observed three years after treatment completion further supports this strategy.

A limitation of this report is that a direct causal relationship between pembrolizumab therapy and sarcoidosis-like reaction could not be definitively established, although the temporal association strongly suggested an immune-related etiology. Additionally, standardized diagnostic and management strategies for immune checkpoint inhibitor-associated sarcoidosis-like reactions have not yet been established. Nevertheless, this case underscores the importance of tissue confirmation when imaging findings are ambiguous and systemic response to therapy is otherwise favorable. This case emphasizes the importance of multidisciplinary evaluation when atypical radiologic findings arise during immune checkpoint inhibitor therapy. Awareness of pulmonary drug-induced sarcoidosis-like reaction is essential to ensure accurate diagnosis and optimal therapeutic decision-making.

## Conclusions

This case shows that pulmonary nodules arising during immune checkpoint inhibitor therapy do not always indicate metastatic progression, even in patients with recurrent uterine carcinosarcoma. When systemic disease remains well controlled, sarcoidosis-like reaction should be included in the differential diagnosis. Tissue confirmation can be particularly valuable when imaging findings are inconclusive, and the diagnosis would alter clinical management.

Awareness of this diagnostic pitfall may help clinicians avoid premature discontinuation of effective immunotherapy or unnecessary escalation of treatment. Careful multidisciplinary evaluation remains essential for appropriate decision-making in similar cases.
